# Impact of Obesity on Outcomes of Patients With Hip Osteoarthritis Who Underwent Hip Arthroplasty

**DOI:** 10.7759/cureus.10876

**Published:** 2020-10-10

**Authors:** Hafeez Shaka, Pius E Ojemolon

**Affiliations:** 1 Internal Medicine, John H. Stroger, Jr. Hospital of Cook County, Chicago, USA; 2 Anatomical Sciences, St. George's University, St. George's, GRD

**Keywords:** obesity, osteoarthritis, hip arthroplasty, surgical outcomes

## Abstract

Background

While obesity has been clearly established as a risk factor for osteoarthritis (OA), there is a scarcity of studies comparing outcomes between obese and non-obese patients with hip OA who underwent hip arthroplasty.

Methods

This study involved adults with hip OA who had hip replacement procedures. Data was sourced from the Nationwide Inpatient Sample (NIS) database for 2016 and 2017. The primary outcome was inpatient mortality. Secondary outcomes included the development of non-ST segment elevation myocardial infarction (NSTEMI), sepsis, post-procedure site infection, pneumonia, acute kidney failure, deep vein thrombosis (DVT), pulmonary embolism, need for transfusion of blood products, complications involving orthopedic devices as well as mean length of hospitalization and mean total hospital charges.

Results

Obese patients did not have higher odds of in-hospital mortality (adjusted odds ratio [aOR]: 0.65, 95% CI 0.303-1.381, p=0.260), had increased mean length of hospitalization (0.11, 95% CI 0.083-0.134, p<0.001) and higher odds of developing DVT (aOR: 1.62, 95% CI 1.187-2.222, p<0.001), acute kidney failure (aOR: 1.64, 95% CI: 1.488-1.807, p<0.001) and pressure-related injuries (aOR: 1.64, 95% CI 1.081-2.483, p=0.020), compared with non-obese patients. Obese patients were found to have a lower aOR of having NSTEMI (aOR: 0.57, 95% CI 0.332-0.986, p=0.044), and need for blood product transfusion (aOR: 0.80, 95% CI 0.726-0.875, p<0.001).

Conclusion

Although there is no difference in mortality among obese and non-obese patients who had hip arthroplasty, obese adults have increased odds of morbidity and perioperative complications. Hence, obese adults likely require better perioperative management to decrease the incidence of these complications.

## Introduction

The World Health Organization defines obesity as abnormal or excessive fat accumulation that presents a risk to health. The most widespread statistical definition of obesity is a body mass index (BMI) of 30 kg/m^2 ^or more. The prevalence of obesity has markedly increased over the last three decades, and this is perpetuated by economic growth, mechanized transport, an increasingly sedentary lifestyle, and a nutritional transition to processed foods and high-calorie diets. It is estimated that by 2030, 20% of the world’s population will be obese [1]. Obesity is one of the most well-defined risk factors for osteoarthritis (OA).

Globally, OA is the most common degenerative disease of the joints, leading to substantial pain and disability world over [2]. After the knees and small joints of the hand, the hips are the most frequently affected joints in OA. Hip OA causes severe pain and stiffness of the hip joints leading to significant disability that often requires surgical intervention. Hip OA is the commonest reason for hip replacement surgery in the United States [3-6]. According to the Global Burden of Disease 2010 study, the global age-standardized prevalence of hip OA was 0.85% (95% uncertainty interval 0.74%-1.02%) [7].

The association between obesity and OA has recently been shown to go beyond increased mechanical stress at joints. Systemic factors such as inflammatory mediators, cytokines, adipokines, growth factors, proteinases, and regulatory mechanisms (including interleukin-1, tumor necrosis factor-alpha [TNF-α], interferon γ, transforming growth factor-beta [TGF-β], matrix metalloproteinases, and reactive oxygen species) have been implicated in the pathogenesis of OA. These systemic factors increase the susceptibility of joints to OA by direct damage to joint tissues or by impairing the process of repair in damaged joint tissues. These factors are also thought to be more relevant in the pathogenesis of hip and hand joint OA than knee OA [3,8-10].

While obesity has been clearly established as a risk factor for OA, there is a scarcity of studies comparing outcomes between obese and non-obese patients with hip OA who underwent hip replacement surgery. The primary aim of this study is to compare the inpatient mortality rates for patients with and without obesity admitted with hip OA who eventually underwent hip replacement using a nationally representative database. The secondary aim of the study is to investigate possible disparities in post-surgical complications between these two patient populations.

## Materials and methods

Design and data source

A retrospective cohort study was carried out among patients with hip OA who underwent hip arthroplasty during the same hospitalization. The Nationwide Inpatient Sample (NIS) database for 2016 and 2017 was used to obtain the study cohort. The NIS database is an administrative dataset containing discharge data to approximate national figures [11-12].

Study population

The study involved adults (aged 18 years and above) with a primary discharge diagnosis of hip OA who underwent any hip replacement procedure. Patients were excluded if they had revision arthroplasty, or if arthroplasty was done during a prior admission. This cohort was further divided based on the presence of at least one secondary discharge diagnosis of obesity, or a BMI greater than 30.

Outcome measures

The primary outcome was inpatient mortality. Secondary outcomes included the odds of having a secondary discharge diagnosis of non-ST segment elevation myocardial infarction (NSTEMI), sepsis, post-procedure site infection, pneumonia, acute kidney failure, deep vein thrombosis (DVT), pulmonary embolism, need for transfusion of blood products, complications involving orthopedic devices as well as mean length of hospitalization and mean total hospital charges.

Statistical analysis

Data analysis was done using Stata® Version 16 software (StataCorp, TX, USA). Analyses were conducted using the weighting samples for national estimates in adjunct with Healthcare Cost and Utilization Project regulations for using the NIS database. A chi-square test was used to compare baseline characteristics and comorbidities between obese and nonobese groups. Outcomes were calculated using multivariate regression analysis to adjust for confounding variables (obtained from a literature review). A univariate screen was done to further confirm whether these factors affected outcomes with variables having a p-value less than 0.2 included in the multivariate regression analysis. The total length of hospital stay and the total hospital charges between the obese and non-obese groups were compared using a multivariate linear regression model. A p-value of <0.05 was set for statistical significance.

Ethical considerations

This study was exempt from Institutional Review Board approval as the database is de-identified and does not contain protected healthcare information.

## Results

Patient characteristics

The combined NIS database for 2016 and 2017 contained over 71 million weighted hospital discharges of which 757,345 satisfied the inclusion criteria for the study. These patients were adults with a principal discharge diagnosis of hip OA who underwent hip replacement procedures. Of this group, just over a fifth (21.8%) were obese, defined by International Classification of Diseases, 10th Revision (ICD-10) codes.

The obese patients were significantly younger (63.2 vs 66.2 years, p<0.001), predominantly Caucasian females, and were majorly insured through Medicaid (48.6%). Obese patients had more comorbidities including hypertension (62.2 vs 50.4%, p<0.001), smoking history (36.4 vs 32.4%, p<0.001), congestive heart failure (3.8 vs 2.1%, p<0.001) and chronic kidney disease (5.8 vs 3.7%, p<0.001) compared with non-obese patients. Patient and hospital characteristics are detailed in Table 1.

**Table 1:** Characteristics of patients with hip osteoarthritis who had hip replacement CHF: congestive heart failure; CKD: chronic kidney disease; CVA: cerebrovascular accident; IHD: ischemic heart disease; COPD: chronic obstructive pulmonary disease. Figures in parentheses are percentages.

Variable	Overall	Non-obese	Obese	p-value
Patient characteristics				
Number	757,345	592,110 (78.2)	165,245 (21.8)	
Mean age (years)		66.2	63.2	<0.001
Women	(55.5)	(55.6)	(55.2)	0.223
Racial distribution				<0.001
White	(82.2)	(82.9)	(79.4)	
Black	(7.2)	(6.5)	(10.1)	
Hispanic	(3.4)	(3.3)	(3.4)	
Others	(7.2)	(7.3)	(7.1)	
Insurance type				<0.001
Medicaid	(55.0)	(56.8)	(48.6)	
Medicare	(4.7)	(4.4)	(5.7)	
Private	(39.7)	(38.2)	(45.1)	
Uninsured	(0.6)	(0.6)	(0.6)	
Charlson comorbidity index score				<0.001
0	(62.8)	(65.6)	(52.6)	
1	(22.8)	(21.6)	(27.2)	
2	(8.5)	(7.8)	(11.3)	
≥3	(5.9)	(5.0)	(8.9)	
Median annual income in patient’s zip code, US$				<0.001
1-43,999	(19.3)	(18.9)	(20.7)	
44,000-55,999	(28.9)	(24.7)	(25.5)	
56,000-73,999	(27.3)	(27.1)	(28.0)	
≥74,000	(28.5)	(29.3)	(25.8)	
Co-morbidities				
Hypertension	(53.0)	(50.4)	(62.2)	<0.001
Smoking history	(33.3)	(32.4)	(36.4)	<0.001
CHF	(2.5)	(2.1)	(3.8)	<0.001
CKD	(4.2)	(3.7)	(5.8)	<0.001
Dyslipidemia	(41.6)	(39.7)	(48.3)	<0.001
Diabetes mellitus	(15.0)	(12.3)	(24.7)	<0.001
Chronic IHD	(10.8)	(10.5)	(12.2)	<0.001
Prior CVA	(0.4)	(0.3)	(0.4)	0.004
Atherosclerosis	(0.5)	(0.5)	(0.7)	<0.001
Liver disease	(1.0)	(0.8)	(1.5)	<0.001
COPD	(6.9)	(6.7)	(7.8)	<0.001
Hospital characteristics				
Hospital region				<0.001
Northeast	(20.4)	(20.1)	(21.5)	
Midwest	(26.1)	(24.9)	(30.3)	
South	(32.4)	(33.1)	(29.8)	
West	(21.1)	(21.9)	(18.4)	
Hospital bed size				0.1453
Small	(29.5)	(29.7)	(29.0)	
Medium	(27.9)	(28.1)	(27.2)	
Large	(42.6)	(42.2)	(43.8)	
Urban location	(92.0)	(91.8)	(92.2)	0.001
Teaching hospital	(64.8)	(64.0)	(67.6)	<0.001

Primary outcome: in-hospital mortality

The in-hospital mortality for patients with hip OA who had a replacement procedure was 0.032% of the total cohort. In total, there were 240 estimated deaths. Obese patients did not have higher odds of in-hospital mortality (adjusted odds ratio [aOR]: 0.65, 95% CI 0.303-1.381, p=0.260) when adjusted for co-morbidities.

Secondary outcomes

Obese patients had increased mean length of hospitalization (0.11, 95% CI 0.083-0.134, p<0.001) compared to non-obese patients. There was, however, no difference in the mean total hospital charge between them. Obese patients were found to have a lower aOR of having a secondary discharge diagnosis of NSTEMI (aOR: 0.57, 95% CI 0.332-0.986, p=0.044), and need for blood product transfusion (aOR: 0.80, 95% CI 0.726-0.875, p<0.001). Obese patient, however, had higher odds of having a secondary discharge diagnosis of DVT (aOR: 1.62, 95% CI 1.187-2.222, p<0.001), acute kidney failure (aOR: 1.64, 95% CI 1.488-1.807, p<0.001), and pressure-related injuries (aOR: 1.64, 95% CI 1.081-2.483, p=0.020) compared to non-obese patients. Detailed outcomes are provided in Table 2.

**Table 2:** Clinical outcomes in hospitalizations comparing non-obese versus obese patients principally admitted for hip osteoarthritis who had hip replacement surgery in the United States from 2016 to 2017 CI: confidence interval; NSTEMI: non-ST segment elevation myocardial infarction. *Statistically significant. ^#^Mean difference.

Outcome	Obese (n=165,245)	Non-obese (n=592,110)	Adjusted odds ratio (95% CI)	p-value
Primary outcome				
In-hospital mortality	40	200	0.65 (0.303 – 1.381)	0.260
Secondary outcomes				
Length of stay, mean days	2.2	2.1	0.11^#^ (0.083 – 0.134)	<0.001*
Total hospital charges, mean US$	60,750	60,551	-386^#^ (-1341 – 568)	0.428
NSTEMI	85	465	0.57 (0.332 – 0.986)	0.044*
Sepsis	150	320	1.34 (0.831 – 2.131)	0.235
Pneumonia	275	825	1.02 (0.738 – 1.400)	0.919
Post-procedure infection	45	105	1.34 (0.538 – 3.359)	0.527
Deep vein thrombosis	305	750	1.62 (1.187 – 2.222)	0.002*
Pulmonary embolism	175	490	1.15 (0.763 – 1.728)	0.506
Complications from orthopedic device	700	2480	0.97 (0.805 – 1.180)	0.793
Need for transfusion of blood products	4610	19,950	0.80 (0.726 – 0.875)	<0.001*
Acute kidney failure	4095	7900	1.64 (1.488 – 1.807)	<0.001*
Pressure-related injury	175	385	1.64 (1.081 – 2.483)	0.020*

## Discussion

Obesity has been strongly associated with an increased risk of OA onset, faster progression, and more severe disability in patients with OA [1,3,11]. According to the Centers for Disease Control and Prevention, the age-adjusted prevalence of obesity (BMI ≥ 30 kg/m^2^) was 42.4% among adults aged 20 and over in the United States in 2017-2018 [13]. This is almost twice as much as the prevalence of obesity in our study population (21.8%) and may point towards deliberate attempts among OA patients to control their weight, as weight loss is the most commonly recommended lifestyle modification for obese patients with OA [14-16]. More than 55% of our study population were females, with similar representation in both the obese and non-obese cohorts. This is supported by several studies that highlight the increased risk of OA in females [3,17,18].

According to our study, obesity was not shown to negatively affect in-hospital mortality rates among patients with hip OA who underwent hip replacement. Analysis of the patient characteristics in our study showed that obesity was associated with a significantly higher risk of co-morbidities such as hypertension, smoking history, congestive heart failure, chronic kidney disease, dyslipidemia, atherosclerosis, liver disease, and chronic obstructive pulmonary disease, which on their own are associated with worse outcomes following hip replacement surgery [19]. After adjusting for these co-morbidities, there was no significant difference in the in-hospital mortality between both the obese and non-obese cohorts. There is a marked paucity of studies that highlight the effect of obesity on in-hospital mortality after hip replacement surgery. However, a population-based cohort study conducted by Tohidi et al., in which the authors followed up patients that had total hip arthroplasty for hip OA between 2002 and 2007, found that morbidly obese patients (BMI > 45 kg/m^2^) were 38% more likely than non-morbidly obese patients to die within 10 years, after adjusting for baseline differences in age, sex, socioeconomic status, and comorbidity [20]. A similar study conducted by Ward et al. in 2015 found that patients who were morbidly obese were over twice as likely to die in the first year following total joint arthroplasty (hip or knee replacement) as non-morbidly obese patients (OR: 2.46, 95% CI 1.34-4.52) [21]. The findings of increased mortality risk in the obese cohorts in these studies may however not directly be related to the effect of obesity on hip replacement surgery outcomes, but may be more in keeping with the increased risk of morbidity and mortality associated with obesity in the general population [22]. This highlights the need for more studies comparing in-hospital mortality between obese and non-obese patients who underwent hip replacement surgery for hip OA.

Our study also demonstrated significantly longer mean hospital length of stay, higher risk of NSTEMI, DVT, acute kidney injury, and pressure-related injuries in the obese cohort compared to non-obese patients. Similar findings were observed in a study conducted by Wallace et al. that showed that while an increased risk of DVT and longer hospital stays were observed among obese patients who had hip replacement surgery, no such association was observed for stroke and mortality [23]. These findings were also buttressed by the outcomes of a prospective cohort study conducted by Haebich et al. [24] and a systematic review conducted by Haynes et al. [25] that showed that obese patients are at an elevated risk of perioperative and post-operative complications when compared with non-obese controls. Additionally, our study showed no difference in mean hospital charges, risk of sepsis, pneumonia, post-procedure infection, pulmonary embolism, or complications from orthopedic devices. This is at variance with findings of a study conducted by Walls et al. that showed that following hip arthroplasty, morbid obesity was independently associated with superficial surgical site infection (OR: 2.02 with 95% CI 1.36-3.02, p<0.01) and the composite endpoint of “any infection” (OR: 1.42 with 95% CI 1.05-1.93, p<0.01) [26]. The effect of confounders like hypoalbuminemia that was significantly more in the obese cohort was however not ruled out and may explain the increased risk of infections in the obese cohort observed in the study.

Our study has several strengths. First, the data was sourced from the NIS, a large nationwide dataset, to provide a large sample size that enabled us to compare mortality outcomes despite the low inpatient mortality rate associated with this cohort of patients. Secondly, the nature of the database allows us to provide insights into the comparison of baseline demographics and hospital outcomes between hip OA hospitalizations with and without concomitant obesity to statistically significant levels.

There are some limitations to the study. First, NIS database studies are subject to non-randomization. Second, there may be coding errors, as the NIS is an administrative database that uses ICD-10 codes to characterize diagnoses and hospitalization events. Third, the NIS database deals with hospitalizations, not individual patients, so patients admitted multiple times will be counted multiple times. Fourth, there is no reliable way to determine if the secondary diagnoses preceded or developed during the index hospitalization. Additionally, laboratory and radiologic data like erythrocyte sedimentation rate, C-reactive protein assay, and hip radiographs that could indicate underlying disease severity and inflammatory activity are not available in the NIS database.

## Conclusions

Although there is no difference in mortality among obese and non-obese patients who had hip arthroplasty, obese adults have increased odds of morbidity and perioperative complications. Hence, obese adults likely require better perioperative management to decrease the incidence of these complications.
